# A Deadly Poison

**Published:** 1875-12

**Authors:** 


A Deadly Poison.—There are several metals which are worth more than "their weight in gold," among them, "Osmium," which sells for $335 per lb., gold being valued at $320. Osmium is the most deadly poison known to chemistry. Twenty pounds of this deadly mineral would be more than enough to destroy the entire population of the earth. One thousandth part of a grain of osmic acid, set free in a volume of air of one hundred cubic yards, would possess such a deadly influence, that all persons respiring this air would be poisoned. What makes it the more dangerous is the fact that it has no antidote. Fortunately, very little of the substance is known to exist.

---

Cleanliness is next to Godliness, and with people who perspire much, is right along side of it.

